# Alda-1 reduces chronic and post-deprivation nicotine intake in rats: impact of concurrent ethanol consumption on treatment efficacy

**DOI:** 10.3389/fphar.2026.1880813

**Published:** 2026-10-13

**Authors:** Fresia Solís-Egaña, Sofía Adasme-Reyes, Ignacio Gutiérrez-Vega, María Elena Quilaqueo, María Elena Quintanilla, David Vásquez, Mario Herrera-Marschitz, Paola Morales Retamales, Mario Rivera-Meza

**Affiliations:** 1 Department of Pharmacological and Toxicological Chemistry, Faculty of Chemical Sciences and Pharmacy, University of Chile, Santiago, Chile; 2 Transdisciplinary Nucleus of Pharmacology and Immunology, ICBM, Faculty of Medicine, University of Chile, Santiago, Chile; 3 Department of Neuroscience, Faculty of Medicine, University of Chile, Santiago, Chile; 4 Specialized Center for Prevention of Substance Use and Treatment of Addictions (CESA), University of Chile, Santiago, Chile

**Keywords:** Alda-1, ALDH2, ethanol, GLT-1, glutamate, nicotine, nucleus accumbens, post-deprivation intake

## Abstract

**Background:**

Nicotine dependence is characterized by high relapse rates, often complicated by comorbid alcohol consumption. Both substances dysregulate glutamatergic signaling and promote oxidative stress in the nucleus accumbens (NAc), mechanisms that may contribute to persistent drug seeking. This study evaluated the therapeutic potential of Alda-1, an activator of mitochondrial aldehyde dehydrogenase 2 (ALDH2), to reduce nicotine and concurrent ethanol/nicotine intake, post-deprivation reaccess, and examined its association with accumbal glutamate homeostasis.

**Methods:**

Female alcohol-preferring UChB rats were induced to voluntarily consume nicotine or a combination of nicotine and ethanol. Rats received Alda-1 (25 mg/kg, p. o.) or vehicle during chronic maintenance and during a 14-day deprivation period. Extracellular glutamate levels in NAc were assessed by, and GLT-1 levels were determined by Western blot.

**Results:**

Alda-1 administration significantly suppressed chronic nicotine intake by 67% and reduced post-deprivation nicotine consumption by 82%. These behavioral changes were accompanied by a 40% reduction in extracellular glutamate levels in the NAc, restoring them to baseline levels observed in naïve controls. In the co-consumption model, Alda-1 effectively reduced chronic ethanol and nicotine intake by 80%–90%. However, Alda-1 failed to reduce post-deprivation intake in the dual-substance paradigm. This lack of efficacy was accompanied by GLT-1 downregulation induced by concurrent ethanol/nicotine exposure, which was not reversed by Alda-1.

**Conclusion:**

Our findings indicate that ALDH2 activation by Alda-1 markedly reduced chronic and post-deprivation nicotine intake, an effect associated with improved glutamate homeostasis. Alda-1 also attenuates chronic polysubstance use; however, its efficacy is lost during post-deprivation re-access in the co-consumption model associated with GLT-1 downregulation, suggesting synergistic neuroadaptations may bypass ALDH2-mediated pathways.

## 1 Introduction

Tobacco use disorder is characterized by high relapse rates and the limited long-term efficacy of current pharmacotherapies (Lee et al., 2021; Zhang et al., 2023; VanFrank et al., 2024; Aubin et al., 2014; Onwuzo et al., 2024). This clinical challenge is severely compounded by comorbid alcohol consumption. Between 80% and 90% of individuals with alcohol use disorders also exhibit nicotine dependence (DiFranza and Guerrera, 1990; Kohut, 2017). Conversely, individuals with nicotine dependence are approximately four times more likely to be dependent on alcohol compared to the general population (Grucza and Bierut, 2006). Because concurrent consumption represents a distinct and highly refractory clinical state (Nguyen et al., 2020), there is an urgent need for novel pharmacological strategies that target neuroadaptations underlying both nicotine and alcohol dependence.

Mechanistically, both alcohol and nicotine converge on activation of the mesocorticolimbic dopamine system, increasing dopamine release from the ventral tegmental area (VTA) to the nucleus accumbens (NAc) (Di Chiara and Imperato, 1988). This sustained activation induces neuroadaptations that contribute to compulsive drug-seeking and relapse, involving, among other systems, glutamatergic neurotransmission (Lüscher and Malenka, 2011; Kalivas, 2009; Scofield et al., 2016). In this context, chronic exposure to alcohol and nicotine has been associated with increased extracellular glutamate levels in the NAc (Griffin et al., 2014; Melendez et al., 2005; Reid et al., 2000; Rossetti and Carboni, 1995). Moreover, whereas nicotine exposure markedly increases accumbal glutamate release in ethanol-dependent animals, this effect is absent in naïve subjects (Saellstroem et al., 2006), suggesting that prior ethanol exposure alters glutamatergic responsivity to nicotine. Collectively, these findings suggest that alcohol–nicotine co-exposure is associated with a dysregulated glutamatergic state in the NAc. This glutamatergic dysregulation has been linked to reduced GLT-1 expression in the NAc (Gipson et al., 2013; Das et al., 2016; Knackstedt et al., 2009), the primary astrocytic transporter responsible for extracellular glutamate clearance and termination of glutamatergic signaling (Kalivas, 2009). Pharmacological restoration of GLT-1 function using ceftriaxone reduces resumption of drug intake in preclinical models of both alcohol and nicotine use (Qrunfleh et al., 2013; Sari et al., 2016), supporting the involvement of impaired glutamate uptake in vulnerability to post-deprivation re-access.

In parallel, chronic exposure to alcohol and nicotine has also been associated with cerebral oxidative stress (Barr et al., 2007; Xu et al., 2019). Crucially, chronic exposure to both substances increases the formation of reactive aldehydes (Bhagwat et al., 1998; Haorah et al., 2008), which can directly impair GLT-1 function and dysregulate glutamate homeostasis (Trotti et al., 1998). Within this context, aldehyde metabolism has emerged as a relevant modulatory pathway (Chen et al., 2014). Alda-1 is a small-molecule activator that acts as an allosteric chaperone; it directly binds to mitochondrial aldehyde dehydrogenase 2 (ALDH2), stabilizing its active conformation and enhancing its catalytic efficiency to rapidly clear reactive aldehydes and reduce oxidative damage (Chen et al., 2014; Fu et al., 2014; Khan et al., 2022). Our laboratory has demonstrated that activating ALDH2 with Alda-1 reduces ethanol acquisition, chronic consumption, and post-deprivation intake in UChB rats (Rivera-Meza et al., 2019; Quilaqueo et al., 2023). Because nicotine and ethanol share overlapping glutamatergic and oxidative neuroadaptations—specifically, the aldehyde-mediated impairment of glutamate clearance—we designed the current study to test whether this modulatory mechanism extends to nicotine and polysubstance use. Therefore, we hypothesized that Alda-1 administration would attenuate chronic and post-deprivation nicotine intake, as well as concurrent alcohol and nicotine consumption, by restoring extracellular glutamate levels and GLT-1 expression in the NAc.

## 2 Materials and methods

### 2.1 Animals

Fifty-eight female Wistar-derived alcohol preferring UChB of approximately 16 weeks old and 150–200 g at the start of the experiments, sourced from the vivarium of the Faculty of Medicine, Universidad de Chile, were used in the study. UChB rats fulfill the key criteria for an animal model of alcohol use disorders (AUD) (Quintanilla et al., 2006; Tampier and Quintanilla, 2010) and have previously been utilized in screening potential drugs for AUD and nicotine abuse (Quintanilla et al., 2008; Sotomayor-Zárate et al., 2013; Quintanilla et al., 2021; Quintanilla et al., 2023). Rats were individually housed in polycarbonate cages in temperature- and humidity-controlled rooms, under a standard 12-h light-dark cycle (lights off at 7:00 p.m.), with food and water available *ad libitum*. Following the experimental procedures, animals were anesthetized with ketamine/xylazine (100/10 mg/kg, i. p.) and euthanized via guillotine decapitation in an auxiliary room. This method was selected for its alignment with the experimental objectives and its compliance with international guidelines (AVMA, 2020) for the euthanasia of rats exceeding 200 g. All animal procedures adhered to ARRIVE guidelines (Kilkenny et al., 2010) and were approved by the Local Institutional Animal Experimentation Ethics Committee, Faculty of Medicine, University of Chile (CBA#1125 FMUCH).

### 2.2 Drugs

Ethanol (Merck) and nicotine ditartrate (Glentham Life Sciences) solutions for drinking studies were prepared daily using tap water. Alda-1 was synthesized in-house as previously described (Rivera-Meza et al., 2019). Alda-1 was suspended in an aqueous solution of Arabic gum 2% for gavage administration. This route of administration was chosen to provide translational clinical relevance compared with injectable routes. The dose, frequency (25 mg/kg; once daily for 7 days), and delivery method were selected based on previous studies demonstrating their effectiveness in reducing voluntary ethanol consumption in rats (Quilaqueo et al., 2023).

### 2.3 Chronic and post-deprivation nicotine consumption

Since rats do not readily consume nicotine orally, thirteen animals were first pretreated with daily intraperitoneal nicotine injections (0.8 mg/kg/day) for 14 days, which promoted a subsequent escalation of voluntary oral nicotine intake when they were offered a 24-h free-choice between water and a nicotine ditartrate solution at progressively increasing concentrations, until consumption stabilized at 55 mg/L (Quintanilla et al., 2018). To establish consumption and overcome taste aversion, nicotine was introduced using a sequential exposure protocol: the concentration started at 5 mg/L and was increased by 5 mg/L every 4–5 days until reaching 55 mg/L. This escalation phase lasted approximately 60 days, followed by approximately 10 days of stable consumption at 55 mg/L prior to treatment. To study the effects of Alda-1 on chronic nicotine consumption, at day 72, animals received one daily oral dose for seven consecutive days of either Alda-1 (25 mg/kg; n = 6) or vehicle (n = 7). Treatment groups were counterbalanced by their average nicotine intake to ensure equivalent baseline consumption across groups. These specific group assignments were maintained throughout the subsequent deprivation and re-access phases. Starting on day 95, nicotine access was stopped to generate a 14-day deprivation period, followed by a 7-day period of nicotine reaccess. Water was always available. To study the effects of Alda-1 on post-deprivation nicotine intake, during the final 14 days of the study (encompassing the end of deprivation and the re-access period), animals again received a single daily oral dose of Alda-1 (25 mg/kg) or vehicle. On the final day of re-access, the animals were euthanized for blood sample collection. Water consumption was expressed as mL/kg/day, and nicotine consumption was expressed as mg of free-base/kg/day. The 14-day deprivation period was established based on previous studies of nicotine withdrawal in UChB rats (Quintanilla et al., 2021).

### 2.4 Chronic and post-deprivation concurrent ethanol/nicotine consumption

Thirteen female UChB rats were administered daily intraperitoneal (i.p.) injections of 0.8 mg/kg nicotine for 14 days to induce voluntary consumption. Following induction, the rats were chronically exposed for 80 days to voluntary 24-h free-choice drinking between a water bottle and a nicotine ditartrate solution bottle at ascending concentrations, until stabilization at 100 mg/L. This was achieved via a sequential exposure protocol starting at 5 mg/L and incrementally increasing by 5 mg/L (up to 30 mg/L) and then 10 mg/L every 4–5 days. The escalation spanned approximately 70 days, followed by 10 days of stable consumption at 100 mg/L. Since UChB rats are genetically predisposed to prefer alcohol, ethanol was introduced only after nicotine consumption was established to prevent animals from exclusively choosing the preferred ethanol solution over the less palatable nicotine solution. On day 81, a third bottle containing 30% ethanol was added. This three-bottle choice was maintained until day 125. The extension of alcohol exposure was on previous studies of alcohol exposure in UChB rats (Quilaqueo et al., 2023). To study the effects of Alda-1 on chronic concurrent nicotine/ethanol consumption, at day 98, animals received one daily oral dose for seven consecutive days of either Alda-1 (25 mg/kg; n = 6) or vehicle (n = 7). Group treatment assignment was counterbalanced based on the average concurrent intake of nicotine and ethanol, and these assignments were maintained throughout the deprivation and re-access phases. Starting on day 126, nicotine and ethanol access were stopped to generate a 14-day deprivation period, followed by a 7-day period of nicotine/ethanol reaccess. To study the effects of Alda-1 on post-deprivation concurrent nicotine/ethanol intake, during the final 14 days of the study (encompassing the end of deprivation and the re-access period), animals again received a single daily oral dose of Alda-1 (25 mg/kg) or the vehicle. On the final day of re-access, the animals were euthanized for blood sample collection. Water consumption was expressed as mL/kg/day, nicotine consumption was expressed as mg of free-base/kg/day, and ethanol intake as g/kg/day.

### 2.5 No net flux glutamate microdialysis

To determine the effects of Alda-1 on accumbal glutamate levels in animals exposed to deprivation after chronic ethanol or nicotine/ethanol, we performed a no net flux microdialysis in freely-moving animals. Two new cohorts of animals were exposed to chronic nicotine (n = 14) or ethanol/nicotine (n = 12) consumption as described before, and Alda-1 or vehicle was administered during the last 7 days of the deprivation period. A naïve group (n = 6) was included as a baseline control that received only water, with no drug exposure, pharmacological treatment, or vehicle gavage. Three days before the end of the withdrawal phase, rats were anesthetized with a mixture of air and isoflurane delivered via a nose-fitted mask and positioned in a Kopf stereotaxic apparatus with the skull aligned. A guide cannula (cat. AT9.14. iC, AgnThos, Sweden) was stereotaxically implanted into the left NAc (AP +1.7 mm, ML +0.7 mm, DV −6.0 mm) (Paxinos and Watson, 1998) and secured to the skull with dental cement. On the day following the end of abstinence, a microdialysis probe 2 mm in length (cat. AT9.14.2, AgnThos, Sweden) was inserted into the NAc via the guide cannula and connected to a microdialysis system (CMA/Microdialysis AB, Stockholm, Sweden) equipped with a perfusion setup. In awake animals, probes were perfused with artificial cerebrospinal fluid (aCSF) followed by increasing concentrations of glutamate (0 μM, 0.5 μM, 1 μM, 5 μM, and 10 μM in aCSF) as previously described (Melendez et al., 2005), at a constant flow rate of 2 μL/min. Microfraction collector sampling (60 μL per sample) was performed every 15 min for 6 h, beginning with a 2 h perfusion with aCSF. Samples were analyzed for glutamate extracellular levels by HPLC (YL9100, Young Lin Instruments, Korea) with fluorometric detection. Relevant parameters can be extracted from microdialysis curves: the slope represents the extraction fraction, and the x-intercept represents the steady-state extracellular neurotransmitter concentration. After completing microdialysis analysis, the animals were euthanized to verify probe placement and collect NAc samples.

### 2.6 Western blot analysis of GLT-1

Proteins present in the NAc samples were mixed with Laemmli buffer (40% glycerol, 8% SDS, 20% DTT, 0.08% bromophenol blue, and 250 mM Tris-HCl buffer, pH 6.8) at a 1:4 ratio and incubated at 100 °C for 10 min. To separate the proteins, SDS-polyacrylamide gel electrophoresis (SDS-PAGE) was performed (9% resolving gel and 5% stacking gel), using the same amount of protein for all samples (15 μg). The electrophoresis was carried out by applying a constant voltage (80 V). Upon completion, the proteins separated in the gel were transferred to a 0.45 μm nitrocellulose membrane, and the successful transfer was verified by Ponceau S staining. To prevent nonspecific antibody binding, the membrane was blocked for 1 h with 5% milk (Bio-Rad) in TBST buffer. Subsequently, the membrane was incubated overnight at 4 °C with a rabbit polyclonal antibody against GLT-1 (PA5-19706, Invitrogen; 1:1,000) or a mouse monoclonal antibody against β-actin (G043, Applied Biological Materials; 1:5,000) as a loading control. Afterward, using TBST buffer, a 15-min wash was performed, followed by four 5-min washes, and the membrane was incubated for 1 hour at room temperature with a goat anti-rabbit antibody conjugated to horseradish peroxidase (1:9,000) or a goat anti-mouse antibody (1:10,000). The membranes were visualized via chemiluminescence using a ChemiScope 3,400 imaging system (Clinx Science Instrument Company) and the SuperSignal West Pico Chemiluminescent Substrate rapid detection kit (34,077, ThermoFisher). Band densities were measured using ImageJ software.

### 2.7 HPLC-MS/MS cotinine levels determination

Since nicotine has a short plasma half-life (∼1 h) in rats, we measured its major metabolite, cotinine (t ½∼7 h) (Onoue et al., 2011), to correlate oral nicotine consumption with systemic exposure. Plasma cotinine levels were measured by HPLC-MS/MS as described previously by Kaisar et al. (2017), with minor modifications. Blood samples were centrifuged at 1,500 g for 10 min at 4 °C to separate the plasma. 100 μL of plasma was mixed with 800 μL of acetanilide 50 ng/mL in acetonitrile (1:8). For protein precipitation, the mixture was centrifuged at 15,000 g for 25 min at 4 °C. The supernatant was concentrated under reduced pressure, resuspended in 100 μL of acetonitrile, and sent to an external service for HPLC-MS/MS analysis. The LOD was 0.5 ng/mL, and the LOQ was 2 ng/mL. Acetanilide was used as an extraction reference, as reported by Massadeh et al. (2009), and the measured cotinine concentration was corrected for acetanilide recovery.

### 2.8 Statistical analysis

The data are presented as means ± SEM. The normality of the data was confirmed using the Shapiro-Wilk Normality Test. Glutamate levels and densitometry for GLT-1 Western blot were analyzed using a one-way ANOVA, followed by the Tukey *post hoc* multiple comparison test. Nicotine, ethanol, body weight, water, and total fluid consumption data were evaluated using a repeated-measures (RM) ANOVA with treatment as the between-subjects factor and ethanol solution or total fluid consumption as dependent variables, followed by Bonferroni’s *post hoc* test. Differences in cotinine plasma and nicotine intake levels were analyzed using a two-tailed paired t-test. All statistical analyses were performed using GraphPad Prism eight software (GraphPad Software Inc.). A p-value of less than 0.05 was considered statistically significant.

## 3 Results

### 3.1 Experiment 1: Effect of Alda-1 on chronic nicotine intake and post-deprivation re-access

In the first experiment, rats underwent a 14-day nicotine priming period, followed by a long-term voluntary oral nicotine intake protocol (Figure 1A). By day 71 of access, the animals reached a stable mean consumption of approximately 2.3 mg/kg/day. To test the efficacy of ALDH2 activation during chronic maintenance, rats were treated daily with Alda-1 (25 mg/kg, i. g.) or vehicle for 7 days starting on day 72. Analysis revealed that Alda-1 administration significantly suppressed chronic nicotine intake compared to the vehicle group (days 72–84, 2-way RM-ANOVA *F*_(1, 11)_ = 36.55; *p* < 0.001), reaching a maximal reduction of 67% on the day following the final dose (day 79; 0.78 vs. 2.73 mg/kg/day, *p* < 0.05, Bonferroni’s test) (Figure 1B). Upon treatment discontinuation, animals from the Alda-1 group gradually increased their nicotine consumption, reaching the level of the vehicle group after 5 days of stopping treatment. Alda-1 administration elicited a reduction in fluid intake of 16.3% compared to the vehicle group (2-way RM-ANOVA, *F*_(1, 11)_ = 58.76; *p* < 0.001), an effect that was reversed upon treatment discontinuation (Figure 1C). Analysis of water consumption showed that Alda-1 did not reduce water consumption, suggesting that the drop in total fluid intake was due to its strong reduction of nicotine solution intake (Supplementary Figure S1A).

**FIGURE 1:**
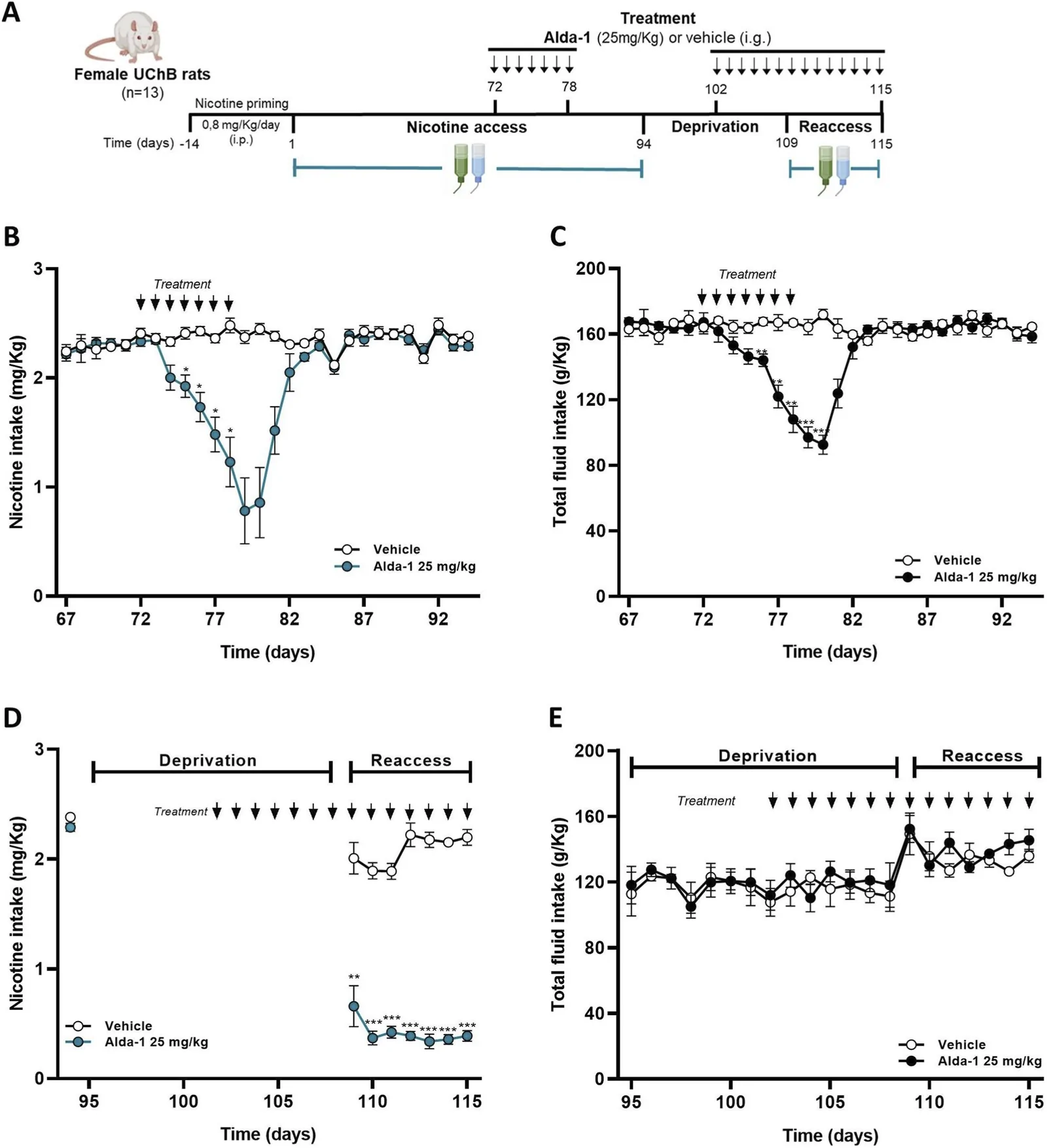
Effect of Alda-1 administration on nicotine chronic intake and post-deprivation re-access. **(A)** Graphical schedule of the experimental design. Thirteen female UChB rats were exposed to a nicotine priming period of 0.8 mg/kg/day for 14 days. The animals were exposed to a long-term 24-h two-bottle free choice paradigm between water and nicotine for 94 days. Finally, in the deprivation phase, the nicotine bottle was removed for 14 days, and then put back on the reaccess phase. Rats were treated (black arrows) daily with Alda-1 (25 mg/kg, i.g.) or vehicle for 7 days, from day 72–78, during the phase of long-term intake, and from day 102–115, during deprivation and reaccess. **(B)** Effect of Alda-1 on chronic nicotine intake. Treatments were administered for 7 days, starting on day 72 (black arrows). White circles represent the vehicle group (n = 7), and blue circles represent the Alda-1 25 mg/kg group (n = 6). A two-way RM ANOVA indicates significant differences between groups (*p* < 0.001). **(C)** Effect of Alda-1 on total fluid intake. Treatments were administered for 7 days, starting on day 72 (black arrows). White circles represent the vehicle group, and black circles represent the Alda-1 25 mg/kg group. A two-way RM ANOVA indicates significant differences between groups (*p* < 0.001). **(D)** Effect of Alda-1 on nicotine intake in the reaccess after deprivation. Treatments were administered for 14 days, starting on day 102 (black arrows). White circles represent the vehicle group, and blue circles represent the Alda-1 25 mg/kg group. The two-way RM ANOVA indicates significant differences between groups (*p* < 0.001). **(E)** Effect of Alda-1 on total fluid intake in reaccess after deprivation. White circles represent the vehicle group, and black circles represent the Alda-1 25 mg/kg group. A two-way RM ANOVA indicates significant differences between groups (*p* > 0.05). (*p < 0.05; **p < 0.01; ***p < 0.001; Bonferroni´s *post hoc* test).

To evaluate the effect of Alda-1 on post-deprivation behavior, nicotine access was suspended for 14 days following the initial 94-day intake period. During this deprivation phase, daily Alda-1 or vehicle treatment began on day 102 and continued through the 7-day re-access period (Figure 1A). Results of Figure 1D show that Alda-1 administration markedly reduces post-deprivation intake (2-way RM-ANOVA *F*_(1, 11)_ = 647.7; *p* < 0.001), achieving a maximal reduction of 82% by the final day of the experiment (0.33 ± 0.04 vs. 1.92 ± 0.08 mg/kg/day, day 115, *p* < 0.001, Bonferroni’s test). Physiological data corroborated this behavioral change, as Alda-1-treated rats exhibited plasma cotinine levels (main metabolite of nicotine) that were 25% lower than those of vehicle-treated animals (2.43 ± 0.03 vs. 3.17 ± 0.19 ng/mL; *p* < 0.001 t-test) (Supplementary Figure S2A). Alda-1 administration did not affect fluid intake compared to the vehicle group (2-way RM-ANOVA, *F*_(1, 11)_ = 0.96; *p* > 0.05) (Figure 1E). Water consumption during reaccess remained stable in the Alda-1 group, whereas a marked decrease was observed in the vehicle group (Supplementary Figure S1B). Pharmacological treatment was not associated with significant changes in body weight across groups throughout the experiment (Supplementary Figure S3A).

To investigate the neurochemical mechanisms underlying the reduction in post-deprivation nicotine intake induced by Alda-1, a new group of rats was exposed to the same schedule as in Experiment 1. However, instead of allowing reaccess to nicotine after deprivation, animals were subjected to cerebral no-net flux microdialysis to determine extracellular glutamate levels in the NAc. Regression analysis of Figure 2A calculated extracellular glutamate concentrations of 9.05 and 5.87 µM for vehicle- and Alda-1-treated animals, respectively. The slope of the regression line representing the extraction fraction (efficiency of recovery) was estimated at 0.12 and 0.22 for vehicle- and Alda-1-treated animals, respectively. For control animals not exposed to nicotine, the calculated extracellular glutamate levels in the NAc were 2.85 µM, with an extraction fraction of 0.12. Results in Figure 2B shows that extracellular glutamate levels in the NAc of the Alda-1-treated group were significantly lower than those found in vehicle treated animals (one-way ANOVA, *F*_(2, 12)_ = 11.55; *p* < 0.01), showing a reduction of 40% (5.74 ± 1.17 vs. 9.29 ± 0.53 µM, *p* < 0.05 Tukey’s test), while no significant differences were found between Alda-1 treated and control (not nicotine exposed) rats (5.74 ± 1.17 vs. 3.35 ± 0.82 µM, *p* > 0.05 Tukey’s test). Probe positioning verification is shown in Supplementary Figure S4A. The levels of the glutamate transporter GLT-1 in the NAc were determined by Western blot. Results in Figure 2C show that no significant differences in the accumbal levels of GLT-1 were detected among the experimental groups (one-way ANOVA, *F*_(2, 17)_ = 1.56; *p* > 0.05).

**FIGURE 2:**
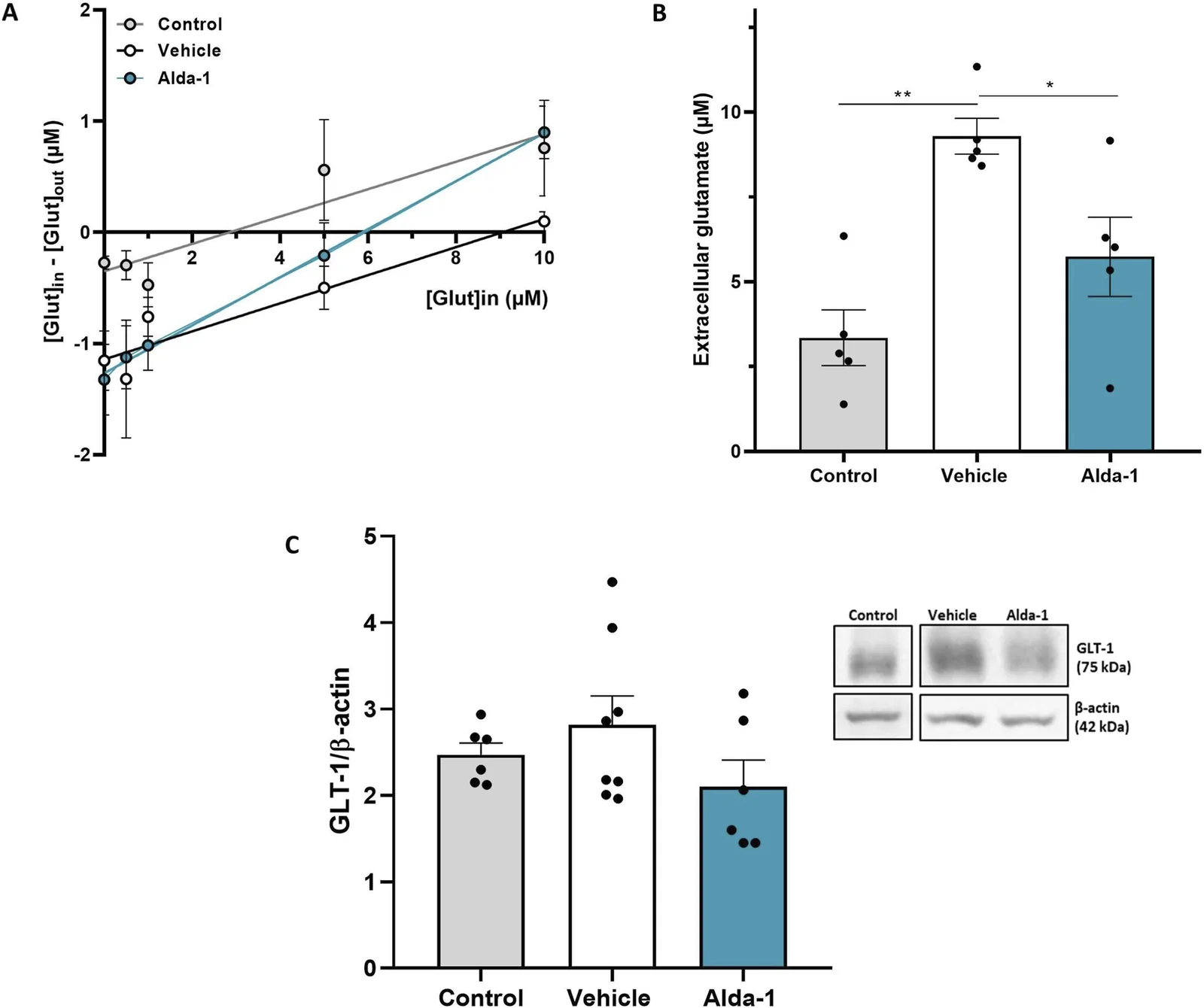
Effect of Alda-1 on glutamate homeostasis in the NAc of rats subjected to deprivation after chronic nicotine consumption. **(A)** Slopes of the regression determined by no net flux microdialysis. The slope of the regression line represents the glutamate extraction fraction. **(B)** Glutamate concentrations in the extracellular fluid of NAc by no net flux microdialysis. Bars represent mean ± SEM of the extracellular concentrations of glutamate in NAc (µM). Gray bar represents control group (n = 5), white bar represents vehicle group (n = 5), and blue bar represents Alda-1 25 mg/kg group (n = 5). A one-way ANOVA test indicates significant differences between groups (*p* < 0.01). Five animals were excluded from glutamate microdialysis analysis due to cannula instability or misplacement. **(C)** GLT-1 expression levels in NAc were determined by Western blot. Bars represent mean ± SEM intensity of the Western blot bands normalized as GLT-1/β-actin ratio. Gray bar represents control group (n = 6), white bar represents vehicle group (n = 8), and blue bar represents Alda-1 25 mg/kg group (n = 6). A one-way ANOVA test indicates no significant differences between groups (*p* > 0.05). (*p < 0.05; **p < 0.01; Tukey´s *post hoc* test). The insert shows representative images from the Western blot analysis of NAc samples. GLT-1 and β-actin images were assembled by cropping original blots. The control group bands were obtained from a different blot than the one used to obtain vehicle and Alda-1 bands (Supplementary Figure S6A).

### 3.2 Experiment 2: Alda-1 effects on concurrent nicotine and ethanol intake

After the initial nicotine priming and 80 days of free-choice nicotine access, ethanol was introduced via a three-bottle free-choice paradigm (Figure 3A). As shown in Supplementary Figure S5, the introduction of 30% ethanol prompted a drop of 18% in chronic nicotine intake. This led to a stable co-consumption state where rats reached mean intakes of 3.62 ± 0.03 mg/kg/day for nicotine and 16.54 ± 0.1 g/kg/day for ethanol. Daily Alda-1 treatment for 7 days (starting on day 98) induced a marked reduction in the consumption of nicotine (days 98–108, 2-way RM-ANOVA *F*_(1, 11)_ = 8.55; *p* < 0.05) (Figure 3B) and alcohol (days 98–108, 2-way RM-ANOVA *F*_(1, 11)_ = 17.22; *p* < 0.01) (Figure 3C). For nicotine, a maximal reduction of 83% was observed (day 103; 0.44 vs. 2.59 mg/kg/day, *p* < 0.05, Bonferroni’s test) while chronic alcohol consumption showed a maximal reduction of 90% on day 104 (1.64 vs. 17.54 mg/kg/day, *p* < 0.05, Bonferroni’s test). Similar to the first experiment, consumption levels for both drugs gradually recovered toward vehicle baselines within approximately 4 days for nicotine and 5–6 days for alcohol following the end of the treatment period. Alda-1 administration did not affect total fluid intake compared to the vehicle-treated group (2-way RM-ANOVA, *F*_(1, 11)_ = 1.76; *p* > 0.05) (Figure 3D). Furthermore, a compensation–like increment in water intake was observed in the Alda-1 group (Supplementary Figure S1C).

**FIGURE 3:**
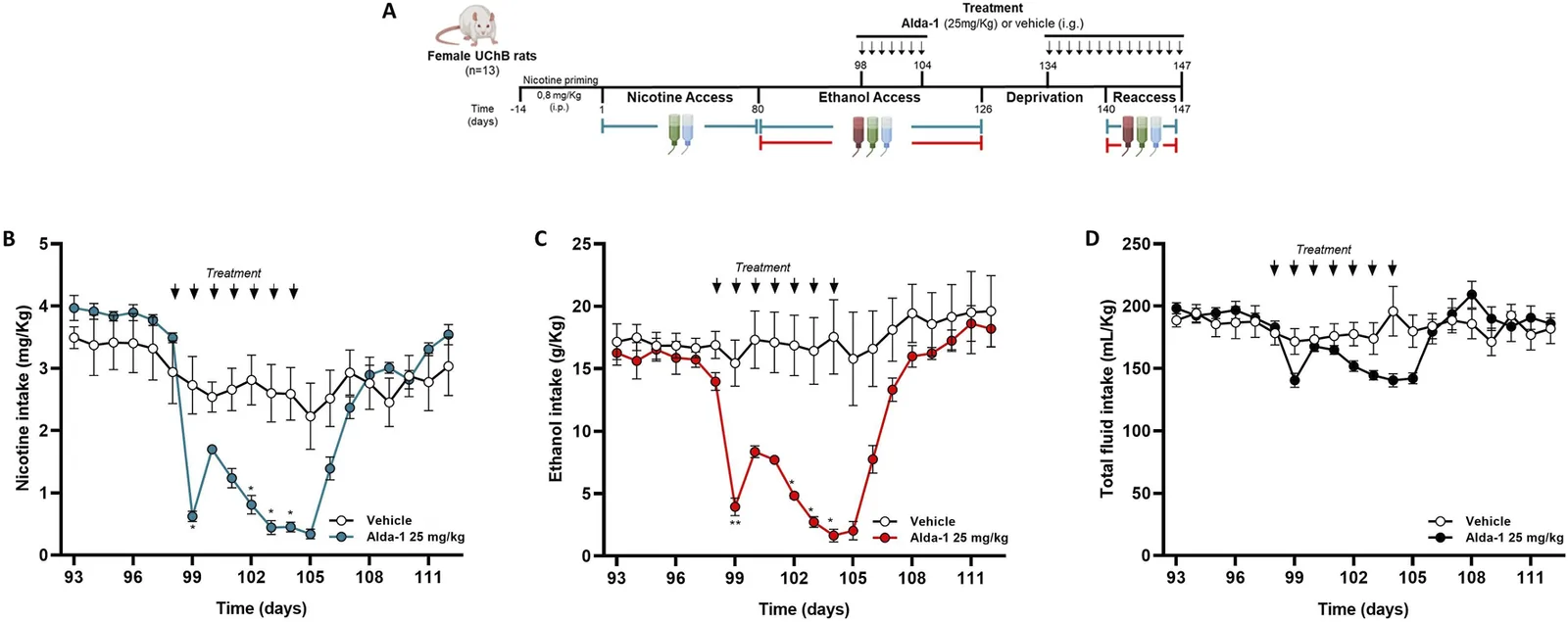
Effect of Alda-1 administration on concomitant nicotine and ethanol chronic intake in UChB rats. **(A)** Graphical schedule of the experimental design. **(B)** Effect of Alda-1 on nicotine intake in a chronic 24-h three-bottle free choice paradigm. Treatments were administered for 7 days, starting on day 98 (black arrows). White circles represent the vehicle group (n = 7), and blue circles represent the Alda-1 25 mg/kg group (n = 6). A repeated-measures two-way ANOVA test indicates significant differences between groups (*p* < 0.05). **(C)** Effect of Alda-1 on ethanol intake in a chronic three-bottle free choice paradigm. Treatments were administered for 7 days, starting on day 98 (black arrows). White circles represent the vehicle group, and red circles represent the Alda-1 25 mg/kg group. A repeated-measures two-way ANOVA test indicates significant differences between groups (*p* < 0.01). **(D)** Effect of Alda-1 on total fluid intake in a chronic three-bottle free choice paradigm. Treatments were administered for 7 days, starting on day 98 (black arrows). White circles represent the vehicle group, and black circles represent the Alda-1 25 mg/kg group. A repeated-measures two-way ANOVA test indicates no significant differences between groups (*p* > 0.05) (*p < 0.05; **p < 0.01; Bonferroni´s *post hoc* test).

Following a 14-day deprivation period for both nicotine and ethanol (days 126–140), the animals were tested for post-deprivation intake while receiving daily Alda-1 or vehicle treatment for 14 days, starting on the eighth day of deprivation and continuing until the end of the experiment (day 147) (Figure 3A). Results from Figure 4A show that the cycle deprivation/reaccess was associated with a generalized reduction in mean nicotine intake, as seen when the vehicle-treated group was compared with the basal intake (1.50 ± 0.05 vs. 2.46 ± 0.24 mg/kg/day, *p* < 0.05, t-test). This effect was not observed for alcohol consumption (15.22 ± 1.35 vs. 15.45 ± 0.36, *p* > 0.05, t-test, Figure 4B). The results also showed that the administration of Alda-1 did not affect, compared with the vehicle-treated group, the concurrent post-deprivation consumption of nicotine (2-way RM-ANOVA, *F*_(1, 10)_ = 0.001; *p* > 0.05, Figure 4A) and alcohol (2-way RM-ANOVA, *F*_(1, 10)_ = 1.83; *p* > 0.05, Figure 4B). The analysis of total fluid consumption showed that Alda-1 administration did not affect fluid intake of the animals compared to the vehicle group (2-way RM-ANOVA, *F*_(1, 10)_ = 0.05; *p* > 0.05) (Figure 4C). Accordingly, water intake was similar between the two experimental groups (Supplementary Figure S1D). Pharmacological treatment was not associated with significant changes in body weight across groups throughout the experiment (Supplementary Figure S3B). This lack of behavioral effect was reflected in the biochemical data, as no significant differences were found in plasma cotinine levels between both groups (4.21 ± 0.46 vs. 5.71 ± 0.95 ng/mL; *p* > 0.05, t-test) (Supplementary Figure S2B).

**FIGURE 4:**
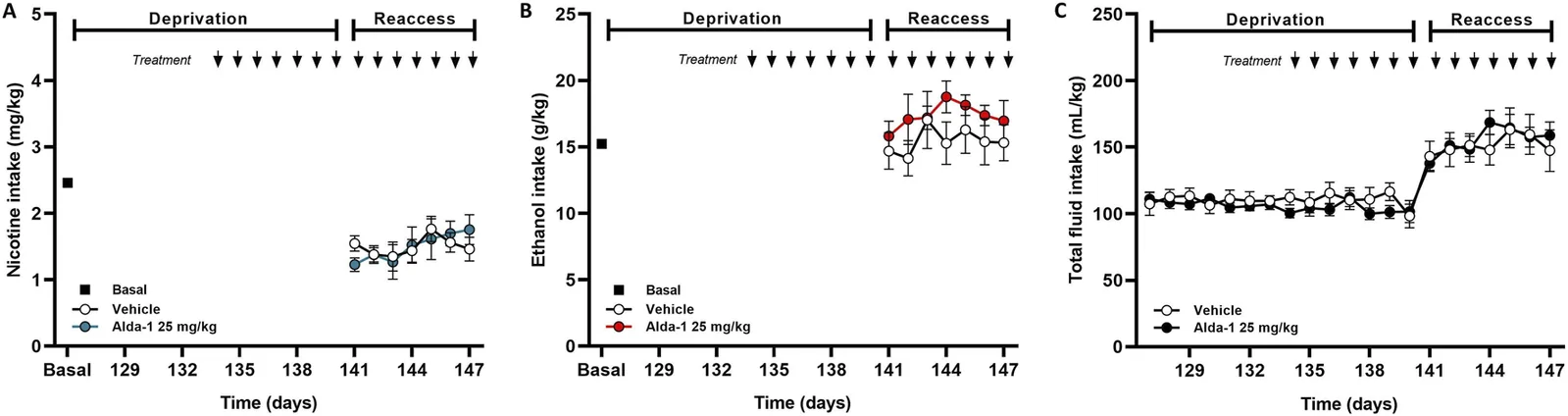
Effect of Alda-1 administration on post-deprivation concomitant nicotine and ethanol consumption in UChB rats. **(A)** Effect of Alda-1 on nicotine post-deprivation intake after a deprivation and reaccess the 24-h three-bottle free choice paradigm. Treatments were administered for 14 days, starting on day 134 (black arrows). White circles represent the vehicle group (n = 7), and blue circles represent the Alda-1 25 mg/kg group (n = 6). The black square represents nicotine baseline consumption prior to the deprivation. A repeated-measures two-way ANOVA test indicates no significant differences between groups (*p* > 0.05). **(B)** Effect of Alda-1 on ethanol post-deprivation intake after a deprivation and reaccess three-bottle free choice paradigm. Treatments were administered for 14 days, starting on day 134 (black arrows). White circles represent the vehicle group, and red circles represent the Alda-1 25 mg/kg group. The black square represents ethanol baseline consumption prior to the deprivation. A repeated-measures two-way ANOVA test indicates no significant differences between groups (*p* > 0.05). **(C)** Effect of Alda-1 on total fluid intake after a deprivation and reaccess three-bottle free choice paradigm. White circles represent the vehicle group, and black circles represent the Alda-1 25 mg/kg group. A repeated-measures two-way ANOVA test indicates no significant differences between groups (*p* > 0.05).

To determine changes in glutamate neurotransmission associated with concurrent nicotine/alcohol intake and pharmacological treatment, a new group of rats was exposed to the same schedule as in Experiment 2, and accumbal glutamate and GLT-1 levels were measured at the end of the deprivation period. Results in Figure 5A show that the no-net-flux regression analysis yields calculated extracellular glutamate concentrations in the NAc of 6.25 and 6.63 µM for vehicle- and Alda-1-treated animals, respectively. The extraction fraction was estimated at 0.28 and 0.29 for vehicle- and Alda-1-treated animals, respectively. For control animals, the calculated extracellular glutamate levels were 2.85 µM, with an extraction fraction of 0.12. Results in Figure 5B showed that no differences in the extracellular glutamate levels in the NAc were found for Alda-1 and vehicle-treated or control animals (one-way ANOVA, *F*_(2, 12)_ = 1.728; *p* > 0.05). Probe positioning verification is shown in Supplementary Figure S4B. Analysis of GLT-1 levels in the NAc (Figure 5C) shows that chronic exposure to concurrent nicotine/alcohol results in a marked reduction in GLT-1 expression compared to naïve control animals (one-way ANOVA, *F*_(2, 15)_ = 13.78; *p* < 0.001). The administration of Alda-1 did not alter GLT-1 levels in the NAc compared with vehicle-treated animals (1.56 ± 0.19 vs. 1.19 ± 0.16, *p* > 0.05, Tukey’s test).

**FIGURE 5:**
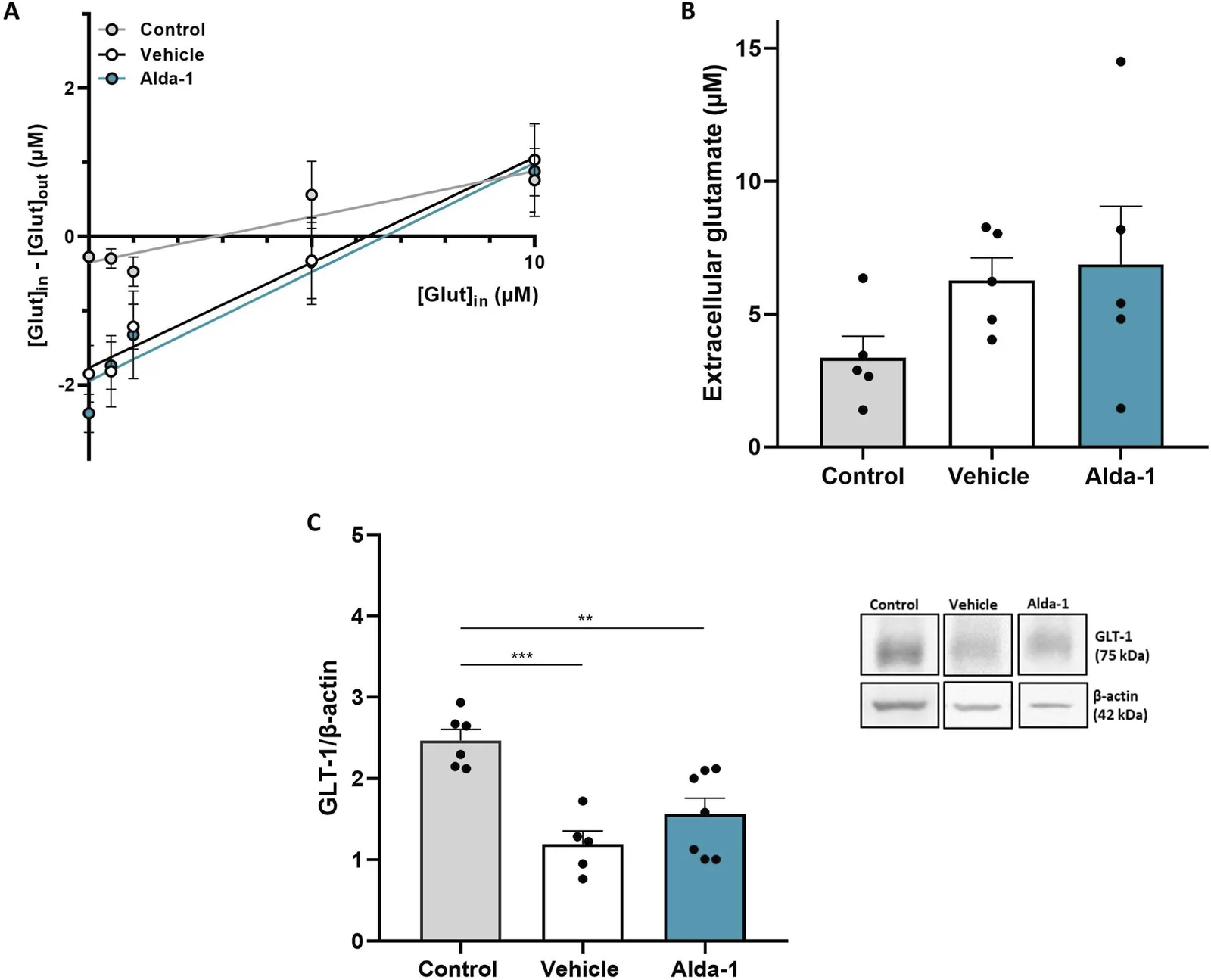
Effect of Alda-1 on glutamate homeostasis in the NAc of rats subjected to deprivation after chronic concomitant nicotine and ethanol consumption. **(A)** Slopes of the regression determined by no net flux microdialysis. The slope of the regression line represents the glutamate extraction fraction. **(B)** Glutamate concentrations in the extracellular fluid of NAc by no net flux microdialysis. Bars represent mean ± SEM of the extracellular concentrations of glutamate in NAc (µM). Gray bar represents control group (n = 5), white bar represents vehicle group (n = 5), and blue bar represents Alda-1 25 mg/kg group (n = 5). A one-way ANOVA test indicates no significant differences between groups (*p* > 0.05). Three animals were excluded from glutamate microdialysis analysis due to cannula instability or misplacement. **(C)** GLT-1 expression levels in NAc were determined by Western blot. Bars represent mean ± SEM intensity of the Western blot bands normalized as GLT-1/β-actin ratio. Gray bar represents control group (n = 6), white bar represents vehicle group (n = 5), and blue bar represents Alda-1 25 mg/kg group (n = 7). A one-way ANOVA test indicates significant differences between groups (*p* < 0.001). (**p < 0.01; ***p < 0.001; Tukey´s *post hoc* test). The insert shows representative images from the Western blot analysis of NAc samples. GLT-1 and β-actin images were assembled by cropping original blots. The control, vehicle, and Alda-1 bands were obtained from different blots (Supplementary Figure S6B). The control (untreated) group is the same as that used for the analysis in Figure 2.

## 4 Discussion

This study demonstrates that Alda-1, an activator of ALDH2, markedly reduces both chronic and post-deprivation nicotine consumption in female UChB rats. These behavioral effects were associated with a significant reduction in extracellular glutamate levels in the NAc, suggesting that Alda-1 may attenuate nicotine-related behaviors, at least in part, by restoring accumbal glutamate homeostasis. When nicotine was consumed concurrently with ethanol, the intake of ethanol was greater than that previously observed when each drug was offered independently in the same rat line. Although Alda-1 significantly reduced chronic concurrent intake, it failed to reduce post-deprivation re-access intake in this dual-substance paradigm. This loss of efficacy was accompanied by an inability of Alda-1 to reverse GLT-1 downregulation or modulate extracellular glutamate levels in the NAc, suggesting that ethanol–nicotine co-exposure induces profound neuroadaptations that are less responsive to ALDH2 activation.

During the chronic phase, UChB rats voluntarily consumed approximately 2.3 mg/kg/day of nicotine, a pharmacologically relevant level consistent with previous oral nicotine intake models (Dadmarz and Vogel, 2003; Kasten et al., 2016). Alda-1 administration significantly suppressed chronic intake by approximately 70%. Interestingly, the inhibitory effects of Alda-1 persisted for several days following treatment cessation. The reasons for this prolonged efficacy remain unclear, particularly considering that Alda-1 has an estimated oral plasma half-life of approximately 8 h (Taneja et al., 2015). One possible explanation is that the high lipophilicity of Alda-1 (LogP = 4.2) may favor its distribution into adipose tissue and other lipid-rich compartments, such as the brain. This tissue retention could allow gradual redistribution into systemic circulation as plasma levels decline, thereby prolonging its pharmacological effect beyond the plasma elimination phase. This interpretation is consistent with the high volume of distribution reported for Alda-1 in rats (41.5 L/kg) and its extensive tissue distribution, both of which are associated with its greater lipophilicity (Taneja et al., 2015). While direct pharmacokinetic evidence confirming that Alda-1 crosses the blood–brain barrier is lacking, its entry into the central nervous system is strongly supported by its high lipophilicity (logP = 4) and previous studies demonstrating brain ALDH2 activation (Fu et al., 2014; Rivera-Meza et al., 2019).

Following a period of forced abstinence, Alda-1 administration also markedly suppressed post-deprivation nicotine consumption by more than 80%. This potent inhibitory effect was superior to previous interventions in comparable rat models, such as N-acetylcysteine or varenicline (Quintanilla et al., 2021; Waeiss et al., 2019). Remarkably, this behavioral effect was accompanied by the normalization of glutamatergic neurotransmission. Chronic nicotine exposure induces significant glutamate dysregulation (Knackstedt et al., 2009; Gipson et al., 2013) and oxidative stress (Barr et al., 2007), thereby impairing GLT-1 function (Trotti et al., 1998). In the current study, Alda-1 restored accumbal glutamate concentrations to levels observed in nicotine-naive control rats and increased the glutamate extraction fraction without altering total GLT-1 protein levels. Chronic nicotine consumption is also associated with elevated cerebral oxidative stress (Barr et al., 2007) and the accumulation of lipid peroxidation-derived aldehydes, such as 4-hydroxy-2-nonenal (4-HNE) (Bhagwat et al., 1998). These reactive species are known to impair GLT-1 function by oxidizing cysteine residues or forming protein adducts (Trotti et al., 1998; Keller et al., 1997; Lauderback et al., 2001). Given that Alda-1 possesses antioxidant properties and accelerates 4-HNE clearance via ALDH2 activation (Singh et al., 2013; Fu et al., 2014), it likely preserves GLT-1 integrity without altering its total expression levels. This model of functional normalization is supported by our observed increase in the extraction fraction alongside stable GLT-1 protein levels in the NAc; however, further mechanistic studies are required to confirm this protective pathway.

Rezvani et al. (2015) previously demonstrated that the ALDH2 inhibitor GS-6637 also reduces nicotine self-administration in rats. Our current finding—that ALDH2 activation also reduces nicotine intake—suggests this enzyme plays a complex regulatory role in the brain’s reward circuitry. Rezvani et al. proposed that inhibiting ALDH2 with GS-6637 leads to elevated levels of tetrahydropapaveroline (THP), a condensation product of dopaldehyde (DOPAL) and dopamine, during nicotine consumption. Because THP inhibits tyrosine hydroxylase, the rate-limiting enzyme in dopamine synthesis (Yao et al., 2010), this THP accumulation likely dampens dopamine release. This diminishes the nicotine-rewarding properties and, consequently, its self-administration. In contrast, we hypothesize that ALDH2 induction by Alda-1 decreases THP generation by accelerating DOPAL elimination following nicotine consumption. This reduction may hamper dopamine release in the brain by attenuating THP-induced µ-opioid receptor activation and DAT inhibition. Further studies are required to establish the precise interplay among ALDH2 activity, dopamine, and THP concentrations, and to determine how these factors mediate the protective effects of both Alda-1 and GS-6637.

In our co-consumption model, UChB rats subjected to the three-bottle free-choice paradigm exhibited mean intakes of 16.4 g/kg/day for ethanol and 3.6 mg/kg/day for nicotine. This level of ethanol intake represents a substantial increase over baseline consumption recorded when it was offered individually (8 g/kg/day) (Quilaqueo et al., 2023). This escalation likely reflects a synergistic interaction between ethanol and nicotine within the NAc dopamine circuitry, as both drugs converge on the same reward pathways via distinct molecular mechanisms (Mansvelder and McGehee, 2002; Israel et al., 2017). Administering Alda-1 during this chronic concomitant phase resulted in a staggering 80%–90% reduction in the intake of both substances. This novel finding highlights the therapeutic potential of ALDH2 activators in mitigating active polysubstance use.

However, Alda-1 failed to modulate consumption levels during post-deprivation re-access in this dual-substance model. Unlike standard single-substance models that typically induce a transient escalation in intake following re-exposure (Martin-Fardon and Weiss, 2013), post-deprivation intake here was comparable to or lower than basal levels, possibly due to taste aversion stemming from the high concentrations of the combined substances during forced abstinence. Regarding accumbal neurochemistry, concomitant ethanol and nicotine exposure induced a generalized downregulation of GLT-1 protein expression in the NAc across all groups. Because Alda-1 failed to reverse this GLT-1 downregulation or normalize extracellular glutamate concentrations at the end of the abstinence period, this persistent glutamatergic dysregulation likely underlies its lack of efficacy in reducing post-abstinence consumption in the co-administration model.

The use of only female rats could constitute a limitation of this study, since it creates a more stringent context for demonstrating the protective effects of Alda-1 against nicotine consumption, because experimental reports have shown that female rats are more prone to self-administer nicotine than male rats (Lynch, 2009; Chellian et al., 2024; Flores et al., 2019). In addition, studies in primary astrocyte cultures have shown that estrogen receptor agonists can induce GLT-1 expression (Lee et al., 2012; Karki et al., 2014), modulating glutamate homeostasis and potentially the *in vivo* response to Alda-1. Further studies in males could confirm these assumptions.

Overall, this study demonstrates that ALDH2 activation via Alda-1 administration significantly attenuates chronic nicotine intake in UChB rats, an effect associated with normalization of extracellular glutamate levels and GLT-1 function, restoring neurochemical homeostasis in the NAc. However, the efficacy of Alda-1 appears to be context-dependent. While it proved highly effective in reducing concurrent intake of alcohol and nicotine during active consumption phases, it failed to reduce its post-deprivation consumption. This lack of efficacy during post-deprivation re-access, coupled with the failure to reverse GLT-1 downregulation in the co-consumption paradigm, suggests that ethanol–nicotine co-exposure may induce persistent neuroadaptations that bypass or overwhelm the ALDH2-mediated pathways. These findings highlight Alda-1 as a promising therapeutic candidate for modulating nicotine-related behaviors while underscoring the need for further research aimed at understanding the distinct neurobiological mechanisms underlying polysubstance use.

## Data Availability

The datasets generated for this study can be found in the Repositorio de datos de investigación de la Universidad de Chile, https://doi.org/10.34691/UCHILE/F2CHON.
