# Supplementary material for: Alda-1 reduces chronic and post-deprivation nicotine intake in rats: impact of concurrent ethanol consumption on treatment efficacy
Source: Front Pharmacol. 2026 Oct 13;17:1880813. doi: 10.3389/fphar.2026.1880813 (PMC13710817; doi:10.3389/fphar.2026.1880813)
Supplement: Supplementary file 1 [file DataSheet1.pdf]

Supplementary Material

1 Supplementary Figures and Tables

1.1 Supplementary Figures

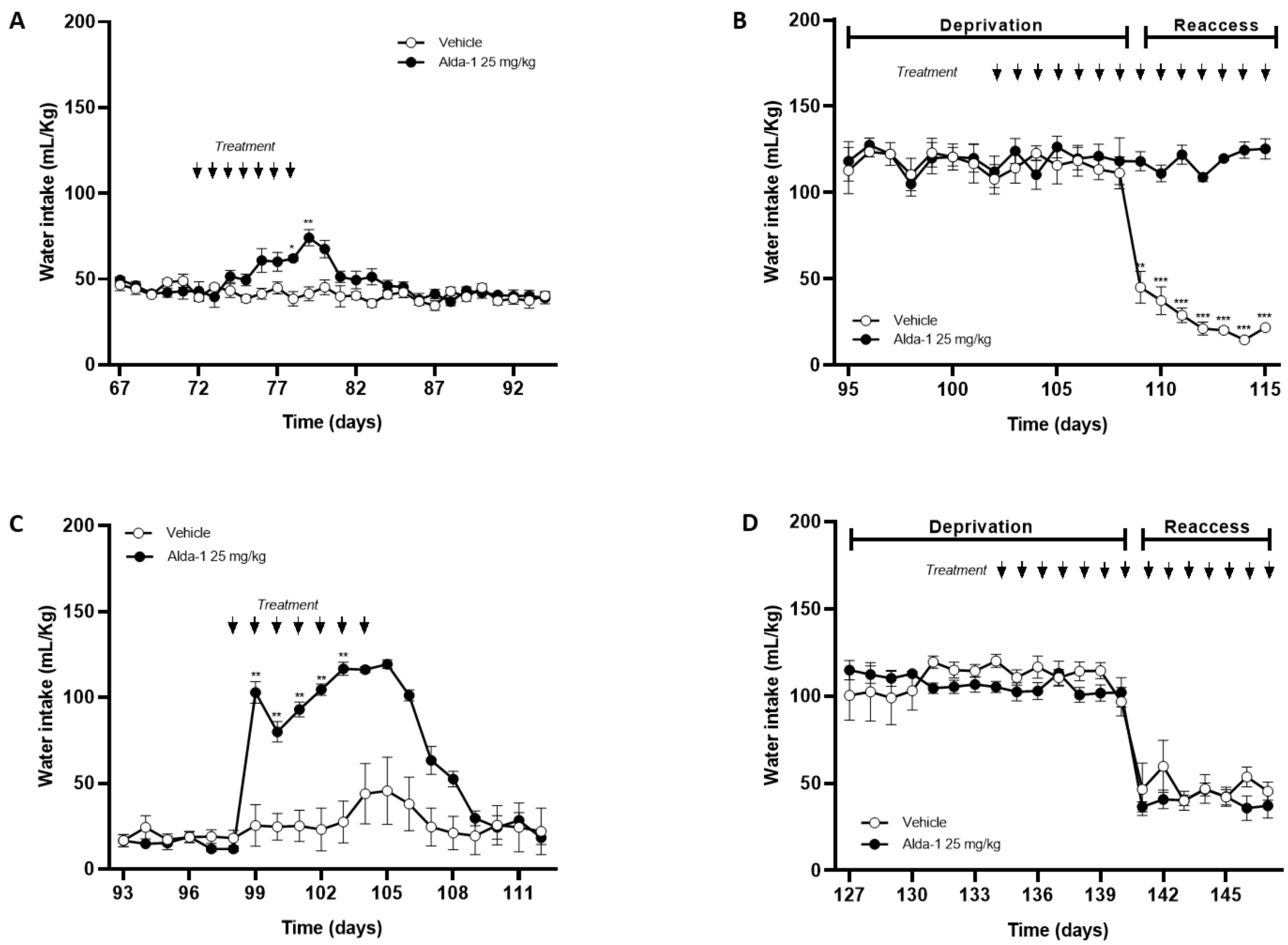

Figure S1. Effect of pharmacological treatment (Alda-1 25 mg/kg or vehicle) on water consumption by rats subjected to chronic and post-deprivation exposure to nicotine or nicotine and ethanol solutions.

(A) Effect of treatment on water intake during chronic exposure to a 24-hour free choice between water and nicotine solution. A two-way RM ANOVA from day 72 to 84 indicates significant differences between groups ($F_{(1, 11)} = 30.79$; $p < 0.001$). **(B)** Effect of treatment on water intake during deprivation and post-deprivation reaccess to a 24-hour free choice between water and nicotine solution. A two-way RM ANOVA indicates significant differences between groups ($F_{(1, 11)} = 92.94$; $p < 0.001$). **(C)** Effect of treatment on water intake during chronic exposure to a 24-hour free choice between water, ethanol, and nicotine solution. A two-way RM ANOVA from day 98 to 108 indicates significant differences between groups ($F_{(1, 11)} = 28.69$; $p < 0.001$). **(D)** Effect of treatment on water intake during deprivation and post-deprivation reaccess to a 24-hour free choice between water, ethanol, and nicotine solution. A two-way RM ANOVA indicates no significant differences between groups ($F_{(1, 10)} = 2.01$; $p > 0.05$). (* $p < 0.05$; ** $p < 0.01$; *** $p < 0.001$; Bonferroni´s post hoc test).

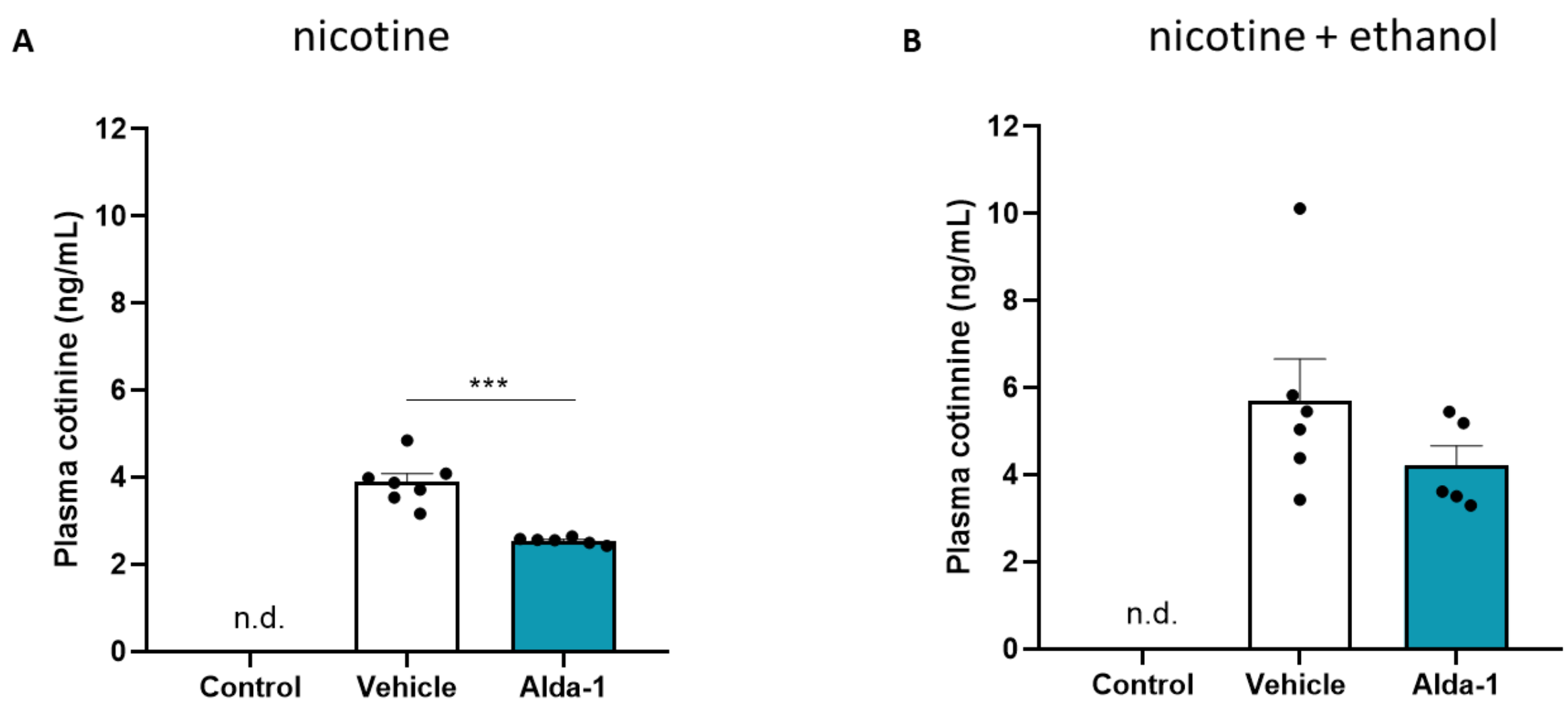

Figure S2. Plasma cotinine concentration of rats exposed to nicotine or nicotine/ethanol deprivation and reaccess.

(A) Plasmatic concentration of cotinine (ng/mL) of rats exposed to deprivation and reaccess in a two-bottle free choice (water/nicotine) paradigm. Bars represent mean ± SEM of the cotinine concentration in plasma. In the control group, cotinine concentration was not detected (n = 6); the white bar represents the vehicle group (n = 7), and the blue bar represents the Alda-1 25 mg/kg group (n = 6). A one-way ANOVA indicates significant differences among groups ($F_{(2,16)} = 238.4$; $p < 0.001$). **(B)** Plasmatic concentration of cotinine (ng/mL) of rats exposed to deprivation and reaccess in a three-bottle free choice (water/nicotine/ethanol) paradigm. Bars represent mean ± SEM of the cotinine concentration in plasma. In the control group, cotinine concentration was not detected (n = 6); the white bar represents the vehicle group (n = 6), and the blue bar represents the Alda-1 25 mg/kg group (n = 5). A one-way ANOVA test indicates no significant differences between groups ($F_{(2,14)} = 23.5$; $p > 0.05$).

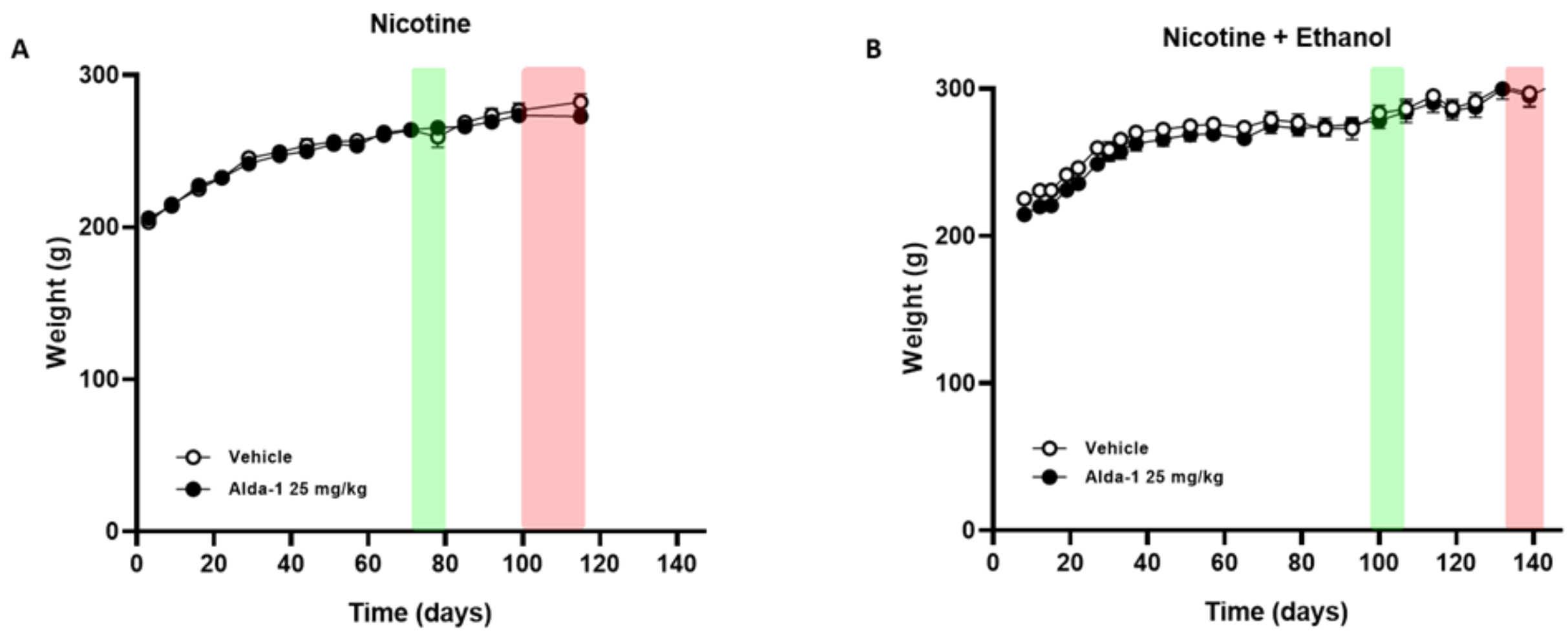

Figure S3. Evolution of body weights of experimental groups. (A) Evolution of body weight of rats subjected to a 24-hour free-choice nicotine intake paradigm. Pharmacological treatments were administered during days 72-78(green rectangle) and 102-115 (orange rectangle). A two-way ANOVA indicated no statistically significant differences across groups ($F_{(1, 413)} = 0.88$; $p > 0.05$). **(B)** Evolution of body weight of rats subjected to a 24-hour free-choice nicotine + ethanol intake paradigm. Pharmacological treatments were administered during days 98-104 (green rectangle) and 134-147 (orange rectangle). A two-way ANOVA analysis indicated a statistically significant difference across groups ($F_{(1, 675)} = 14{,}86$: $p < 0.001$), however a Bonferroni´s *post hoc* test indicated no individual differences.

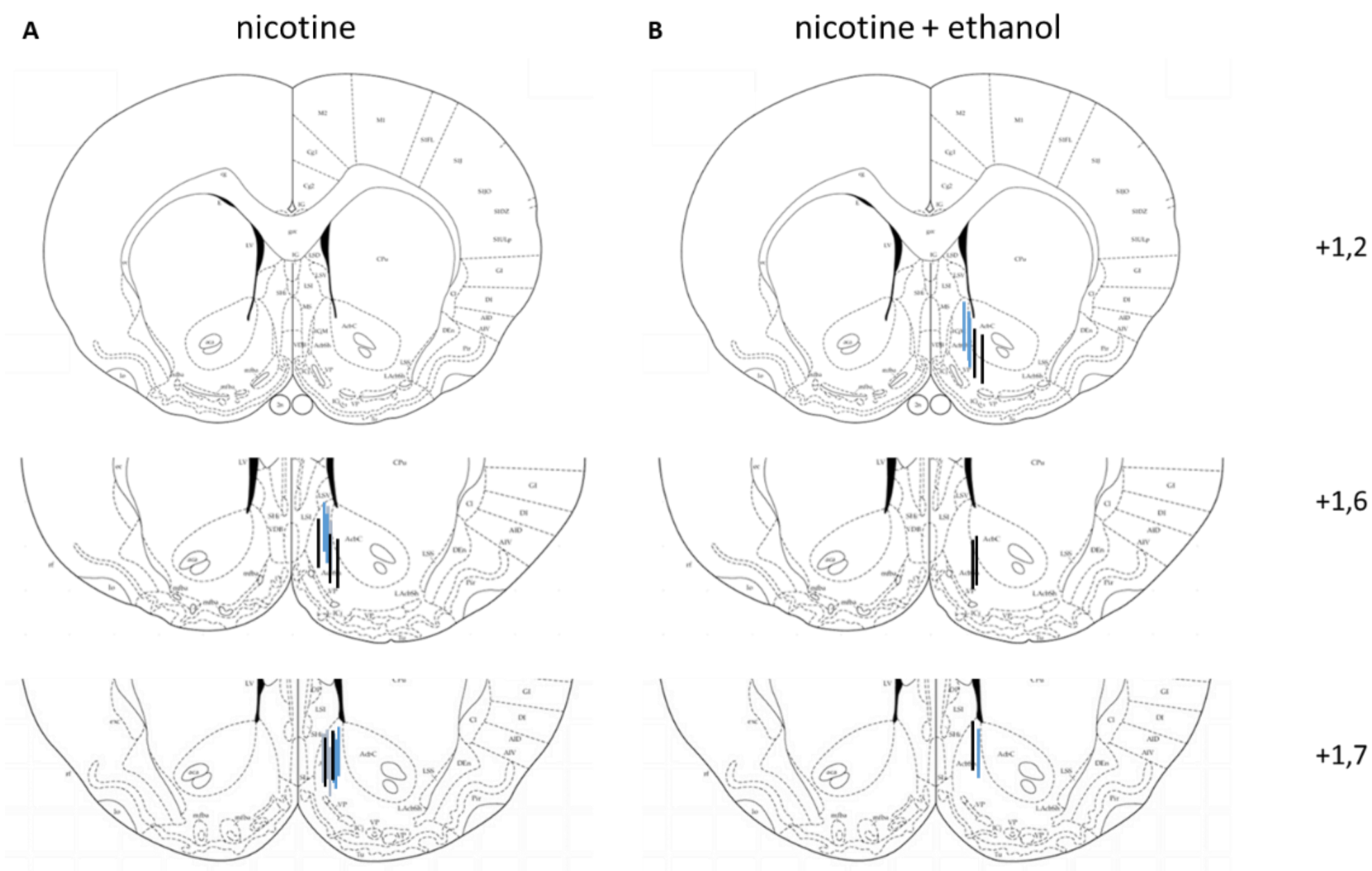

Figure S4. Localization of microdialysis probes.

Guide cannulas were implanted according to the coordinates of the NAc (AP 1.7 mm; L −0.7 mm; DV −6.0 mm; Paxinos G & Watson C, 1998). The diagrams represent the marks left by the guide cannulas in the brain tissue. Lines represent the track left in the tissue by the microdialysis probe that protrudes 2 mm from the end of the guide cannula (Gray: control group; Black: vehicle-treated group; Light blue: Alda-1-treated group). **(A)** Experiment 1: Effect of Alda-1 on glutamate homeostasis in the NAc of rats subjected to deprivation after chronic nicotine consumption. **(B)** Experiment 2: Effect of Alda-1 on glutamate homeostasis in the NAc of rats subjected to deprivation after chronic concomitant nicotine and ethanol consumption. The positions of microdialysis probes in control animals are depicted only in **(A)**.

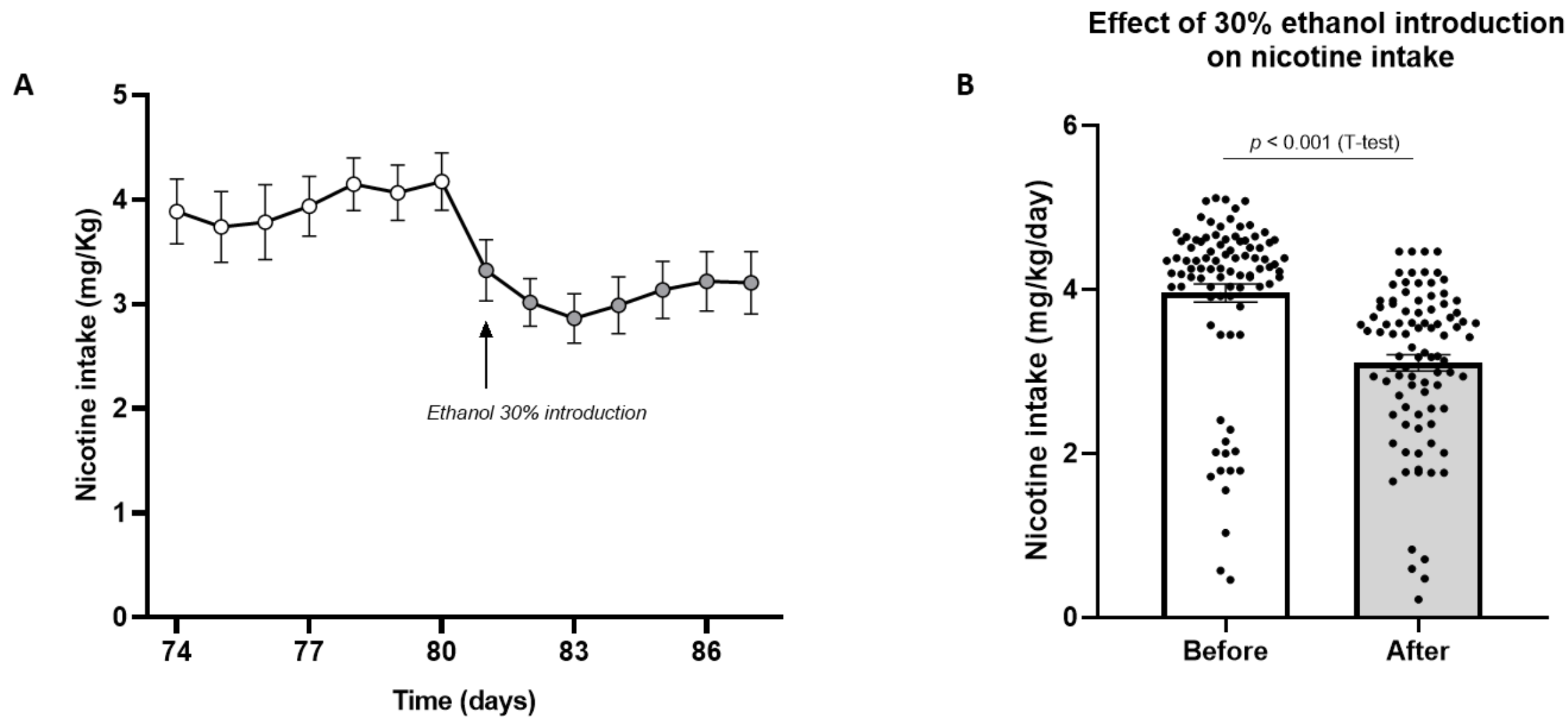

Figure S5. Effect of ethanol introduction on voluntary nicotine consumption. (A) Time-course of daily nicotine intake (mg/kg/day) in female UChB rats 7 days before and 7 days after the introduction of 30% ethanol on day 81 (indicated by the arrow) in the co-consumption paradigm. **(B)** Mean nicotine intake 7 days before and 7 days after the introduction of 30% ethanol. The addition of 30% ethanol to the free-choice paradigm of nicotine and water resulted in a 18% reduction of nicotine intake (t=5.728, df=180; p< 0.001; T-test).

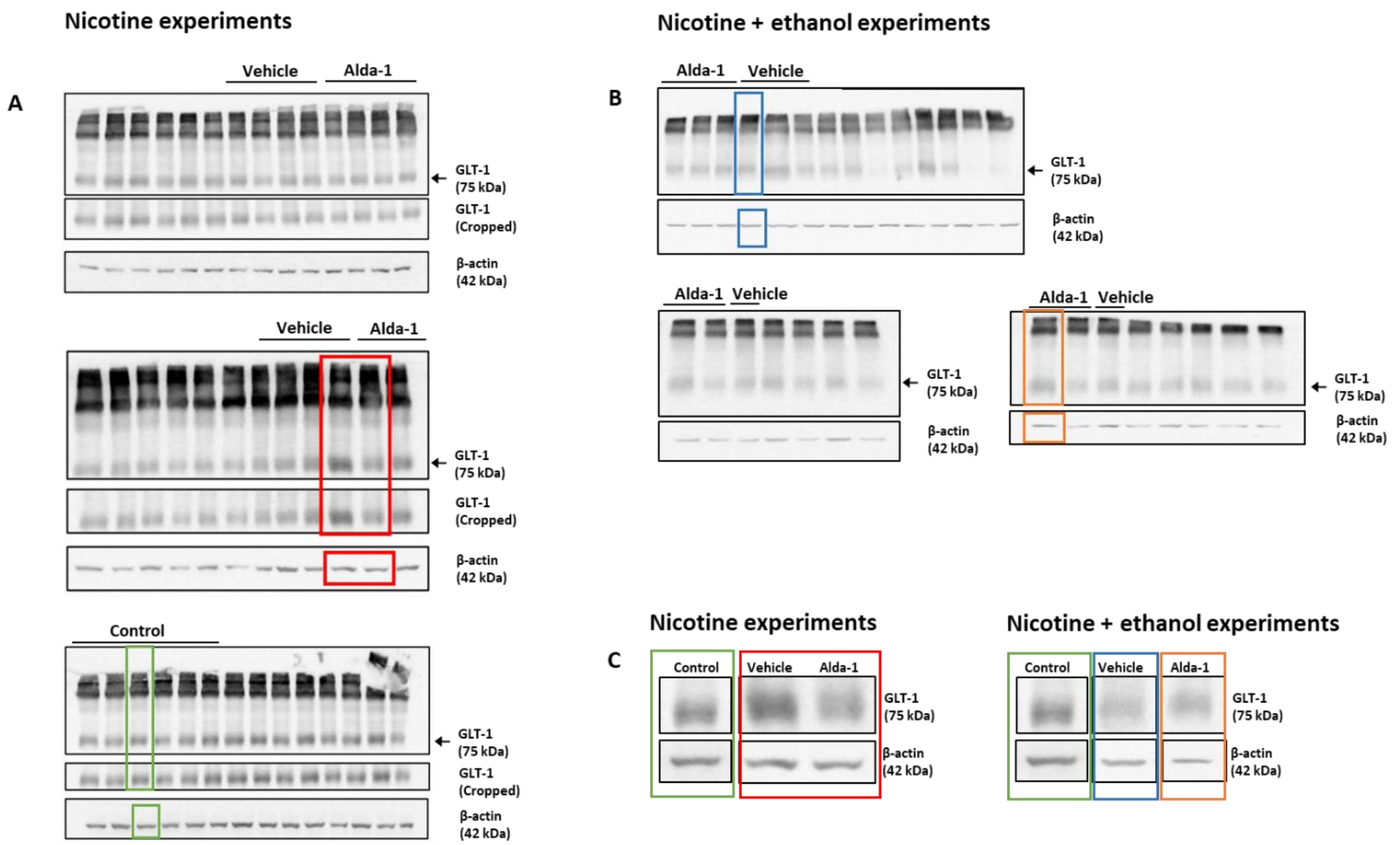

Figure S6. Unprocessed Western blot images and representative bands selection. Unprocessed images for all Western blot data presented in the main manuscript. Original chemiluminescent captures are shown for each biological replicate. The molecular weight of proteins is indicated on the right side of each membrane. No nonlinear adjustments have been applied. Contrast and brightness were adjusted uniformly across the entire image, only where necessary to improve the visibility of the bands and background. **(A)** Effect of Alda-1 on glutamate homeostasis in the NAc of rats subjected to deprivation after chronic nicotine consumption. **(B)** Effect of Alda-1 on glutamate homeostasis in the NAc of rats subjected to deprivation after chronic concomitant nicotine and ethanol consumption. The bands for GLT-1 and β-actin in the control group animals are shown in **(A)**. **(C)** Representative bands selected. For the nicotine experiment (A), representative bands for GLT-1 and β-actin were cropped from the vehicle and Alda-1 groups on the same blot (red square), whereas a different blot was used for the control group (green square). For the nicotine plus ethanol experiments (B), representative bands for GLT-1 and β-actin were cropped from different blots for vehicle (blue square) and Alda-1 (orange square) groups. Control group bands were from the same blot described in (A).
